# A Potential “Anti-Warburg Effect” in Circulating Tumor Cell-mediated Metastatic Progression?

**DOI:** 10.14336/AD.2023.1227

**Published:** 2023-12-27

**Authors:** Zhuofeng Jiang, Jiapeng He, Binyu Zhang, Liping Wang, Chunhao Long, Boxi Zhao, Yufan Yang, Longxiang Du, Weiren Luo, Jianyang Hu, Xin Hong

**Affiliations:** ^1^Department of Biochemistry, School of Medicine, Southern University of Science and Technology, Shenzhen, Guangdong, China.; ^2^Key University Laboratory of Metabolism and Health of Guangdong, Southern University of Science and Technology, Shenzhen, Guangdong, China.; ^3^Guangdong Provincial Key Laboratory of Cell Microenvironment and Disease Research, Southern University of Science and Technology, Shenzhen, Guangdong, China.; ^4^Department of Oncology, Southern University of Science and Technology Hospital, Shenzhen, Guangdong, China.; ^5^Cancer Research Institute, The Second Affiliated Hospital of Southern University of Science and Technology, Shenzhen Third People's Hospital, National Clinical Research Center for Infectious Diseases, Shenzhen, China.

**Keywords:** Circulating tumor cell (CTC), Anti-Warburg Effect (AWE), Metabolic reprogramming, Oxidative phosphorylation (OXPHOS), Metastasis

## Abstract

Metabolic reprogramming is a defining hallmark of cancer metastasis, warranting thorough exploration. The tumor-promoting function of the "Warburg Effect", marked by escalated glycolysis and restrained mitochondrial activity, is widely acknowledged. Yet, the functional significance of mitochondria-mediated oxidative phosphorylation (OXPHOS) during metastasis remains controversial. Circulating tumor cells (CTCs) are considered metastatic precursors that detach from primary or secondary sites and harbor the potential to seed distant metastases through hematogenous dissemination. A comprehensive metabolic characterization of CTCs faces formidable obstacles, including the isolation of these rare cells from billions of blood cells, coupled with the complexities of *ex vivo*-culturing of CTC lines or the establishment of CTC-derived xenograft models (CDX). This review summarized the role of the "Warburg Effect" in both tumorigenesis and CTC-mediated metastasis. Intriguingly, bioinformatic analysis of single-CTC transcriptomic studies unveils a potential OXPHOS dominance over Glycolysis signature genes across several important cancer types. From these observations, we postulate a potential "Anti-Warburg Effect" (AWE) in CTCs—a metabolic shift bridging primary tumors and metastases. The observed AWE could be clinically important as they are significantly correlated with therapeutic response in melanoma and prostate patients. Thus, unraveling dynamic metabolic regulations within CTC populations might reveal an additional layer of regulatory complexities of cancer metastasis, providing an avenue for innovative anti-metastasis therapies.

## INTRODUCTION

Cancer metastasis remains a major clinical obstacle, largely due to its intrinsic heterogeneities, as well as our limited knowledge of the underlying molecular and genetic mechanisms driving disease progression [1-3]. Circulating tumor cells (CTCs) have emerged as an important area of research focus in cancer biology and serve as crucial biomarkers for the non-invasive monitoring of therapeutic responses [4, 5]. Originating as rare tumor cells sloughed into the bloodstream from primary or metastatic loci, CTCs are considered precursor cells that give rise to distant metastases [6-8]. Invasive tumor cells can enter the bloodstream through a process called intravasation, migrate long distances within the blood circulation, colonize distant organs through extravasation, and form metastases that eventually lead to treatment failure and patient death [9, 10]. The intricate biological mechanisms underlying CTC-mediated metastasis remain largely uncharted, primarily attributed to the exceptional scarcity and notably low viability of CTCs. Compounding this challenge are the tremendous technological hurdles associated with the isolation of rare viable CTCs from billions of blood cells within the circulation and their subsequent long-term propagation through *ex vivo* culturing, which are necessary for conducting in-depth experimental investigations [11-13].

Neoplastic cells frequently undergo profound metabolic shifts in comparison to their neighboring normal counterparts, a phenomenon recognized as metabolic reprogramming. This phenomenon manifests prominently across a wide array of documented cancer types, standing as a distinctive hallmark [14]. Metastatic cancer cells, in particular, exhibit remarkable metabolic diversities and remodeling processes that could potentially be influenced by the complex tumor microenvironment present at various stages of the metastatic cascade [15-17]. This intricacy might also hold true for CTCs navigating the complex fluid milieu of the bloodstream.

Upon disengagement from their primary sites, CTCs likely embark on adapting their metabolic pathways to counteract detachment-induced anoikis, a specialized form of apoptosis triggered by disruptions in cell-matrix interactions [18]. Furthermore, they must confront the need to endure fluid-induced shear stresses and a multitude of other challenges while in circulation. Upon successful extravasation from the bloodstream, CTCs may engage in distinct metabolic reprogramming endeavors to prime themselves for effective colonization. Within this paradigm, a metabolic blueprint geared towards overcoming microenvironment-driven metastatic dormancy appears to be of paramount importance [19, 20]. Unfortunately, the scope of available CTC experimental systems, such as CTC cell lines or CTC-derived animal explants (CDX), remains relatively restricted. Consequently, the specific metabolic programs requisite at discrete stages of CTC-mediated metastatic progression and the precise mechanisms driving them remain obscure.

Elevated glycolysis stands as an important hallmark distinguishing cancer cell from their normal counterparts. While glucose metabolism in normal and/or non-proliferating cells largely relies on mitochondrial oxidative phosphorylation (OXPHOS) for ATP production, proliferating cancer cells are mainly dependent on aerobic glycolysis that results in increased lactate production and reduced mitochondrial OXPHOS activities, a phenomenon known as the “Warburg Effect” [21, 22]. Although the "Warburg Effect" is less energetically efficient than the TCA cycle-driven OXPHOS in terms of ATP per glucose unit, it dramatically accelerates glucose uptake and the subsequent production of various glycolytic intermediates that may address the biosynthetic needs of rapidly proliferating cancer cells [23, 24]. The "Warburg Effect" emerges as a pivotal adaptive mechanism to sustain the rapid growth and proliferation of cancer cells, and its role in tumorigenesis and metastatic progression across diverse cancer types is well-established [24-26]. Nevertheless, whether this metabolic trait persists in CTCs remains to be clarified.

In this context, we summarized the impact of the "Warburg Effect" on tumorigenesis and metastasis. Through bioinformatic analysis of publicly available transcriptomic datasets encompassing CTCs and matched primary tumor and/or metastatic cells, an intriguing observation was identified: CTCs derived from melanoma, prostate cancer, lung adenocarcinoma and breast cancer exhibited significantly augmented enrichment of OXPHOS pathway genes over glycolysis when contrasted with corresponding tumor counterparts. These unexpected findings prompted us to formulate the concept of the "Anti-Warburg Effect" (AWE) within CTCs. The observed upregulation of mitochondrial OXPHOS signature in CTCs may represent a potential metabolic switch during the transition of cancer cells from primary tumors to metastatic sites.

## MAIN TEXT

### Technological hurdles in CTC isolation and *ex-vivo* culturing

The systematic characterization of CTCs at the genetic, molecular, and functional levels could only be possible when there is a sufficient number of isolated viable CTCs to start with. This appears to be a substantial challenge given the scarcity and poor survival rates of CTCs upon isolation. The existing CTC isolation technologies are mainly based on the differences in surface protein expression, morphology, physical and biological properties between CTCs and normal blood cells, which could be divided into antigen-dependent and antigen-independent isolation platforms [10, 27].

Antigen-dependent isolation technology utilizes either the positive enrichment of tumor antigen-expressing CTCs or the principle of negative depletion of blood cells using leucocyte-specific surface markers. The positive immunoaffinity enrichment of epithelial cancers is often based on the variable surface expression of epithelial cell adhesion molecules (EpCAM) or cytokeratins (CK) in CTCs [28]. The limitation of positive selection is the potentially biased selection of CTC subpopulations, as CTCs may dynamically alter the molecular phenotype under certain conditions that may lead to the loss of surface marker expressions. The negative depletion methods generally target leucocyte antigens (such as CD45 and CD66a/b) expressed on the surface of white blood cells and enable robust depletion of contaminating immune cells during CTC isolation [29]. The tumor antigen-dependent CTC isolation method may potentially affect CTC viability and cause cellular damage due to direct antibody coating. The antigen-independent isolation method separates CTCs based on physical properties such as size, density, and membrane charge [30, 31]. Various devices using filtrations, density gradient centrifugation, or microfluidic chip-based systems have been developed to capture CTCs with variable detection sensitivities, specificities and efficacies [32-35]. The recent development of the microfluidic CTC-iChip platform by the Haber/Mehmet team at the Massachusetts General Hospital is a fine example of the integration of antigen-independent sorting coupled with negative depletion technologies [36, 37]. Utilizing lateral displacement and inertial focusing-based sorting, and magnetophoresis-based negative depletion strategy, the CTC-iChip is capable of isolating rare viable CTCs from whole blood of multiple different cancer types with high efficiency.

**Figure 1.**
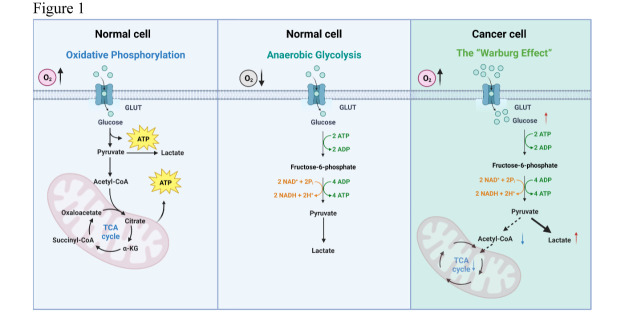
**The glycolysis and mitochondrial oxidative metabolism in normal cells and cancer cells**. Schematics of the distinct metabolic pathways comparing oxidative phosphorylation (OXPHOS) and anaerobic glycolysis in normal cells, and aerobic glycolysis (the “Warburg Effect”) in cancer cells. Glucose is imported by glucose transporter proteins (GLUT), after which crucial enzymes within the glycolytic pathway come into play, facilitating the transformation of glucose into pyruvate. In normal cells, under aerobic conditions, pyruvate is transformed into acetyl coenzyme A (Acetyl-CoA) and subsequently enters the tricarboxylic acid [40] cycle for mitochondria-mediated OXPHOS. In contrast, pyruvate is predominantly converted into lactate even under aerobic conditions in cancer cells. The “Warburg Effect” describes the phenomenon in cancer cells that, independent of oxygen levels, pyruvate is preferentially channeled to support the synthesis of lactate, rather than the production of Acetyl-CoA for mitochondria-mediated OXPHOS.

The CTC cell lines capable of long-term propagation could not only could not only serve as powerful tools for dissecting molecular mechanisms of CTC-mediated metastatic progression, but also are valuable resources for the development of noninvasive tumor biomarkers and potential therapeutic targets [13, 38]. However, expanding a few CTCs into a cell line *ex vivo* has proven to be a challenging task. It has been reviewed elsewhere that there were only about thirty-nine CTC cell lines established successfully from patients bearing different cancers by 2021, including thirteen cell lines from breast cancer, eleven from colon cancer, five from small cell lung cancer, four from malignant melanoma, two from prostate cancer, two from gastroesophageal cancer and two from non-small cell lung cancer [39]. The success rate in establishing CTC cell lines ranging from 1% to 17% [38, 39]. Many factors may affect the expansion of cultured CTC cells, such as CTC isolation platforms, the starting number of CTCs, culture conditions (hypoxia or normoxia atmosphere, culture in 2D with adhesion plate, 3D in suspension or 3D in Matrigel), compositions of culture medium, and other experimental manipulation conditions, with a standard culturing protocol yet to be established across different cancer types [11, 12].

Therefore, the further optimization and development of CTC isolation technologies and CTC *ex-vivo* culturing methods are critically important for propelling CTC biological discoveries and their clinical applications as liquid biopsy.

### The glycolysis in normal and cancer cell

Glycolysis is a complex series of biochemical transformations that convert glucose into pyruvate. In normal cells, the process begins with the transportation of extracellular glucose across the cell membrane via the glucose transporter GLUT1. Once inside the cell, the enzyme hexokinase (HK1/2) initiates the conversion of glucose into glucose-6-phosphate (G-6-P). G-6-P can then undergo reversible conversion into fructose-6-phosphate (F-6-P). Further along the pathway, F-6-P is enzymatically converted into fructose-1,6-bisphosphate (F-1, 6-BP) by the phosphofructokinase-1 (PFK-1). Then, a series of reversible reactions transform F-1,6-BP into phosphopyruvate (PEP). The enzyme pyruvate kinase (PK) mediates the conversion of PEP into pyruvate. The metabolism of pyruvate can take two potential paths: when abundant oxygen molecules are present, pyruvate is converted into Acetyl-CoA through the pyruvate dehydrogenase complex (PDC) to enter the tricarboxylic acid [40] cycle for oxidative phosphorylation (OXPHOS), or alternatively,when the oxygen level in the microenvironment appears low, pyruvate is transformed into lactate through the action of lactate dehydrogenase (LDH), a process that is known as the anaerobic glycolysis [41] (Fig. 1). The biochemical steps of glycolysis controlled by these key metabolic enzymes produce intermediate metabolites to satisfy the needs for macromolecular biosynthesis including nucleotides, lipids, proteins and more. For example, the production of pentose phosphate and NADPH in the pentose phosphate pathway (PPP) requires the conversion of glucose-6-phosphate (G-6-P) from hexokinase during glycolysis [42], which also supplies a large proportion of the reducing equivalents necessary for lipid synthesis [43].

To support rapid growth and division, cancer cells often take a different path for glycolysis, that the pyruvate is mainly converted into lactate, even in the presence of high oxygen levels, which is also known as the “Warburg Effect” or aerobic glycolysis (Fig. 1). Aerobic glycolysis is a low-yielding means of ATP generation (one glucose molecule produces only 2 moles of ATP), far below the energy production of complete oxidation. However, glycolytic ATP production is much faster per unit of time than complete oxidation [24], provided that the supply of glucose is sufficient. Thus, even though the mitochondrial OXPHOS activity is not upregulated in cancer cells, aerobic glycolysis is still fully capable of maintaining the energy balance in tumor cells. Furthermore, it was reported that cancer cells with a high rate, but low yield of ATP production may obtain a selective advantage when nutrient resource in the tumor microenvironment is limited [24, 44, 45]. The “Warburg Effect” has long been a focus in the field of cancer metabolism and appears to be a common phenomenon observed in many cancer types. As reviewed extensively elsewhere in the literature, the molecular and metabolic characteristics associated with the “Warburg Effect” can be summarized in the following: (1) A significant increase in glucose uptake and glycolytic flux; (2) The ability to rapidly produce ATP to maintain energy homeostasis; (3) The rapid synthesis and replenishment of glycolytic intermediates to flow into different metabolic pathways to complete biosynthesis; (4) Restrained oxidative phosphorylation flux by preventing pyruvate from entering the TCA cycle that leads to reduced ROS accumulation; (5) Enhanced lactate production and secretion to remodel tumor microenvironment [14, 46, 47].

### The role of the “Warburg Effect” in the initiation of tumorigenesis

It remains debatable if the “Warburg Effect” is a causative factor of neoplastic transformation. Nevertheless, recent studies have highlighted the significant contribution of the “Warburg Effect” during cancer initiation. In a study using transgenic KRAS^G12D^ pancreatic mouse models, researchers have shown that the stimulation of glycolysis and channeling of glycolytic intermediates into hexosamine biosynthesis and pentose phosphate pathways are major effector mechanisms driving KRAS^G12D^-dependent pancreatic cancer initiation and development [50]. Similarly, the activation of ARF6 induced by KRAS promotes the “Warburg Effect” and PDAC proliferation [51]. During brain cancer development, it was shown that the translocation of PGK1 is directly regulated by oncogenic KRAS and BRAF signalling, which leads to PDHK1 phosphorylation and the inhibition of pyruvate (PDH) complex, and consequently results in enhanced glycolysis and tumorigenesis [52]. In addition to the genetic and molecular alterations within a cancer cell, the occurrence of tumorigenesis is frequently triggered by the unfavourable microenvironment such as hypoxia and nutrient deficiencies [53]. To adapt to the relatively harsh microenvironment and achieve successful outgrowth, neoplastic clones often reprogram their metabolic network into the mode of aerobic glycolysis [53, 54]. This notion is supported by the work demonstrating that a harsh microenvironment in ductal carcinoma in situ (DCIS) of breast cancer selects for the “Warburg Effect” phenotype through transcriptional activation of KLF4, which results in aggressive clonal expansion [55].

Taken together, the metabolic remodeling events leading to enhanced “Warburg Effect” appear to be a consequence of the evolutionary selection of oncogenic clones and may significantly contribute to cancer development in the early stages.

### The role of the “Warburg Effect” effect in cancer metastasis

During metastatic progression, the detached cancer cells must survive against harsh environmental stress. A prominent one is the oxidative stress induced by excessive reactive oxygen species (ROS) production, which may subsequently lead to anoikis [18]. Studies have shown that the “Warburg Effect” plays a critical role in suppressing ROS stress and promoting metastasis. Specifically, the “Warburg Effect” may restrain mitochondrial oxidative metabolic flux by upregulating PDH kinases (PDKs) to phosphorylate and inhibit the key enzyme PDH, which is central for catalyzing pyruvate into acetyl-CoA through the rate-limiting oxidative decarboxylation, and the subsequent entry into TCA cycle. By doing so, the “Warburg Effect” may largely reduce oxidative metabolism and mitochondrial ROS production and protect the disseminating cancer cells from anoikis [56, 57].

The epithelial-to-mesenchymal transition (EMT) is a fundamental characteristic of metastatic progression and the EMT regulators serve as attractive targets for anti-metastasis therapies [58]. The different energetic requirements between mesenchymal and epithelial cells have led to the activation of distinct metabolic pathways to support this transition. It was demonstrated that cancer cells surrounded by certain environmental conditions including hypoxia and low pH levels appeared to exhibit the Warburg Effect, and such phenotypes were related to EMT and cancer metastasis [59]. Studies have shown that certain glycolytic enzymes could indeed regulate the process of EMT. For example, Dong et al. have revealed that the loss of fructose-1,6-bisphosphatase (FBP1) induces glycolysis and enhances glucose uptake, which is a key oncogenic event triggering EMT [60]. In one study, Sun et al. showed that the reduced expression of Pyruvate Dehydrogenase Kinase 4 (PDK4) underlies the metabolic rewiring during EMT [61]. Moreover, recent work has shown that the increase in glycolytic substrates could also accelerate the process of EMT. Under higher glucose concentrations, breast cancer cells exhibit a more invasive phenotype and undergo EMT transition [62]. Certain transcription factors appear to promote EMT by boosting the Warburg Effect. Xu et al. have shown that EGF promotes EMT through the EGFR/PI3K/HIF-1α axis by enhancing glycolysis [63]. In another study on cholangiocarcinoma, the authors found that WDR5 could activate EMT by enhancing HIF-1α transcription and glycolysis through the augmentation of Myc-dependent metabolic remodeling [64]. Therefore, the intricate molecular crosstalk between the Warburg Effect and EMT could be critical drivers of cancer metastasis.

Another critical role of the “Warburg Effect” in promoting metastasis is thought to be the protection against immune surveillance [26, 65]. The tumor microenvironment directly affects metastatic potency [26]. The secreted metabolites produced by cancer cells undergoing glycolysis, including lactate and pyruvate, can induce immune suppression in the local microenvironment [66, 67]. For example, due to the “Warburg Effect”, large quantities of lactic acid are secreted by cancer cells into the tumor microenvironment, which potently inhibits the activation of NK cells and T cells by suppressing the upregulation of nuclear factor of activated T cells (NFAT) [54]. Another study demonstrated that the lactic acid secreted by tumor cells may induce the expression of vascular endothelial growth factor and the M2-like polarization of tumor-associated macrophages, thus creating an immune-suppressive microenvironment [68]. It has also been shown that the secretion and accumulation of lactate within the tumor microenvironment are important for effectively modulating the activation and antigen expression of dendritic cells [69]. Consistently, therapeutic targeting of the lactate transporter gene, MCT, results in the inhibition of tumor growth and invasion [70].

In summary, the “Warburg Effect” has been demonstrated as a metastasis-promoting mechanism. It may achieve this at least in three ways: (1) Limits ROS production by restraining mitochondrial activity and oxidative phosphorylation, thus the disseminated cancer cells are protected from detachment-induced anoikis; (2) Activates EMT through metabolic remodeling to support cancer invasion and metastasis (3) Suppresses immune system activation or modifies the tumor-immune microenvironment through the secretion of glycolytic intermediates from cancer cells, which then allows successful immune evasion and metastatic colonization.

**Figure 2.**
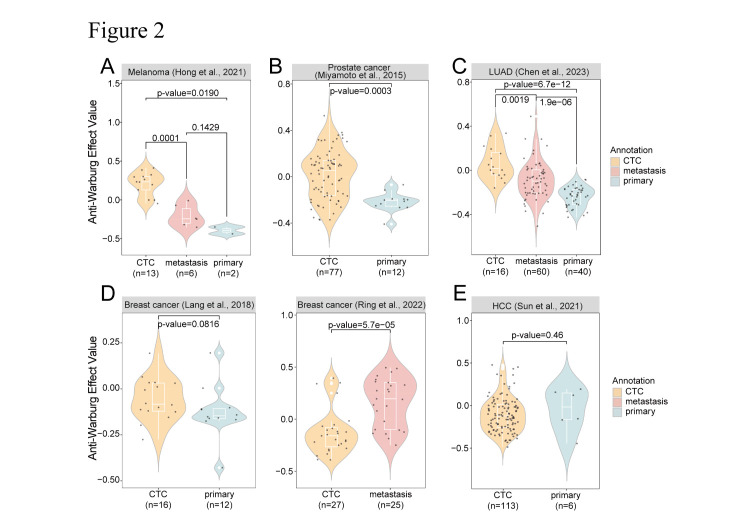
**The evaluation of the potential “Anti-Warburg Effect” (AWE) in CTCs**. Violin plots analyzing the AWE value among CTCs, primary tumors and/or metastases samples in (A) melanoma: CTC(n=13) vs metastasis(n=6): p-value=0.0001; CTC(n=13) vs primary(n=2): p-value=0.0190, (B) prostate cancer: CTC(n=77) vs primary(n=12): p-value=0.0003, (C) lung adenocarcinoma: CTC(n=16) vs metastasis(n=60): p-value=0.0019; CTC(n=16) vs primary(n=40): p-value=6.7e-12 and (D) breast cancer: CTC(n=27) vs metastasis(n=25): p-value=5.7e-05; CTC(n=27) vs primary(n=12): p-value=0.0816, (E) HCC: CTC(n=113) vs primary(n=6): p-value=0.46. The AWE was defined by the enhanced oxidative phosphorylation over glycolysis. The “Anti-Warburg Effect Value” was quantified using the enrichment score of HALLMARK_OXIDATIVE_PHOSPHORYLATION minus the enrichment score of HALLMARK_GLYCOLYSIS. Statistical comparisons in violin plots were accomplished using the Wilcoxon test, with visualization achieved through R packages ggplot2, RColorBrewer, and ggpubr. LUAD: Lung Adenocarcinoma; HCC: Hepatocellular Carcinoma.

### The observation of the potential “Anti-Warburg Effect” in CTCs

It could be speculated that CTCs in circulation are subjected to detachment-induced ROS stress and hence are prone to anoikis. Given the observations that the “Warburg Effect” is effective in limiting OXPHOS and ROS production, we tested if CTCs exhibit a metabolic signature reminiscent of the “Warburg Effect”, or probably behave in the opposite direction that we named the “Anti-Warburg Effect” (AWE) of CTCs. Although the scarcity of viable CTCs isolated from patient blood samples has imposed significant limitations on comprehensively characterizing the metabolic properties of these cells, recent progress in CTC isolation, *ex-vivo* culturing, and single-cell molecular profiling technologies, has provided a significant biological overview of CTC-mediated metastasis [8]. With these advancements, we've conducted a systematic bioinformatic analysis on CTC transcriptomes. A search with the following key words was conducted in the GEO database (www.ncbi.nlm.nih.gov/gds/?term=): CTC[All Fields] OR circulating tumor cell[All Fields] AND "Expression profiling by high throughput sequencing"[Filter]. A total of 106 datasets were accessed, of which 6 datasets were eligible for this study. The gene reads count matrixes of CTCs and tumor tissues were downloaded from the GEO database (GSE157740, GSE113890, GSE198291, GSE67980, GSE111842), GNSA database (CNP0000095) and normalized as log-rank FPKM (Fragments Per Kilobase Million). The H (hallmark gene sets) library was accessed through Molecular Signatures Database (MSigDB) (www.gsea-msigdb.org/gsea/msigdb/human/genesets.jsp?collection=H). Enrichment scores of HALLMARK_OXIDATIVE_ PHOSPHORYLATION and HALLMARK_ GLYCOLYSIS were calculated using R package GSVA with default options (method = ‘gsva’). The AWE Value was defined by the enhanced oxidative phosphorylation over glycolysis which quantified using their enrichment scores (enrichment score of HALLMARK_ OXIDATIVE_PHOSPHORYLATION minus enrichment score of HALLMARK _GLYCOLYSIS). For comparing The AWE Values among CTCs, primary tumors and/or metastases, the function stat_compare_means (method = ‘wilcox.test’) in R package ggplot2 was employed. Single-cell RNA sequencing data were processed with R package Seurat. Malignant cells were annotated with CopyKAT. R packages Seurat, ggplot2, RColorBrewer, ggpubr were used for visualization.

**Figure 3.**
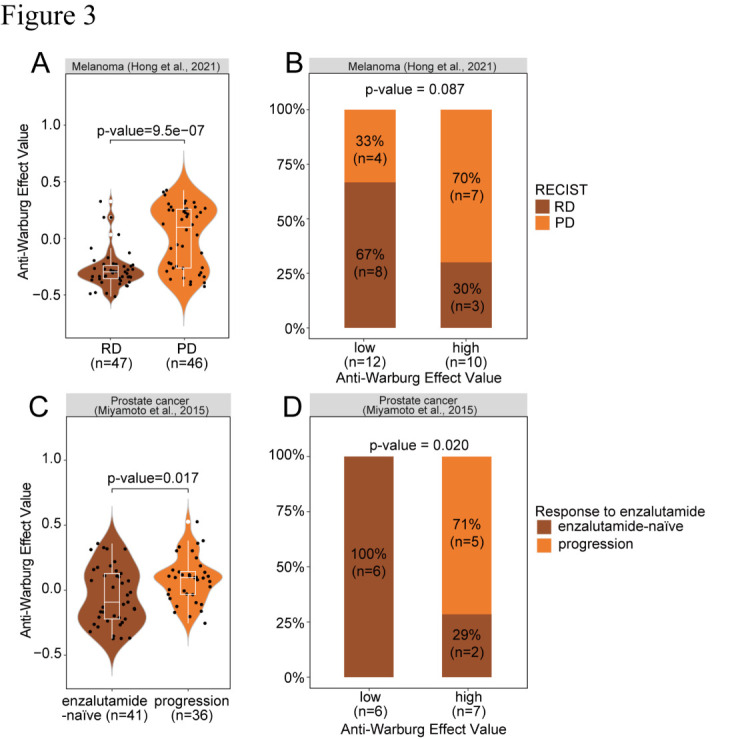
**The correlation between the “Anti-Warburg Effect” (AWE) and therapeutic response**. Violin plots analyzing the AWE among patients with progression and non-progression in (A) melanoma: RD(n=47) vs PD(n=46): p-value=9.5e-07 and (C) prostate cancer: enzalutamide-naïve(n=41) vs progression(n=36): p-value=0.017. In violin plots, each dot represents a CTC. Bar plots displayed the percentage of progression and no progression in patients with high or low AWE value in (B) melanoma: p-value=0.087 and (D) prostate cancer: p-value=0.021. Statistical comparisons in violin plots and bar plots were accomplished using the Wilcoxon test and Fisher’s exact test, respectively. Visualization was achieved through R packages ggplot2, RColorBrewer, and ggpubr. RD: Responding on treatment as defined by RECIST1.1; Progressing on treatment as defined by RECIST1.1.

These single-cell transcriptomic analyses identified an intriguing metabolic trend that we named the “Anti-Warburg Effect” (AWE): when adjusted for glycolysis, the OXPHOS signature expression appeared markedly exceeded that of their primary tumor counterparts in CTCs isolated from the blood samples of melanoma [83], prostate cancer [87], lung adenocarcinoma [86], (Fig. 2A-C). A similar trend was also observed in breast CTCs as compared to primary tumors, although statically it was not significant (P = 0.086, Fig. 2D). Such effect was not observed in hepatocellular carcinoma (HCC), suggesting HCC CTCs may possess a distinct mechanism of metabolic remodeling (Fig. 2E) [84, 85, 88]. These metabolic characteristics observed in CTCs derived from the available cancer datasets are clearly in sharp contrast to the well-recognized “Warburg Effect”, which is marked by upregulated glycolysis and restricted OXPHOS. These results suggested the existence of a unique subset of CTCs, characterized by their heightened expression of OXPHOS over glycolysis signatures across several important cancer types. Notably, in the context of melanoma CTCs, experimental evidence has demonstrated the capability of these cells to proficiently generate tumors and multi-organ metastases in immunocompromised mice [83]. The preferential enrichment for OXPHOS signature may suggest a functionally important role of AWE during CTC survival and/or metastatic colonization.

In order to estimate the correlation between AWE values and disease progression, firstly, we compared AWE values in CTCs between RD/enzalutamide-naïve and PD/progression. The significance was estimated with Wilcoxon test. In addition, patients were defined as low- and high- AWE groups according to the AWE values of their CTCs (cutoff: mean value of all CTCs). Statistical analysis for the correlation between the response to drugs and low/high AWE was performed with Fisher’s exact test.

The correlation between the AWE Values of CTCs and disease progression status were calculated in both melanoma and prostate cancer patients, from which the clinical treatment information was available [83, 87]. Interestingly, the AWE Value of individual CTCs was significantly higher in the patient’s group with progressing disease (PD) as compared to patients with responding disease (RD) in melanoma (P = 9.5e-7, Fig. 3A) [83]. In a separate analysis, the AWE Value of each patient was defined with the calculated mean of the AWE Value of his/her CTCs. The patients were then grouped into high VS low groups based on the comparison to the median AWE value of all CTCs. We found there was a trend between high-level AWE value and disease progression in melanoma (P = 0.087, Fig. 3B). Such correlation was also observed in prostate cancer, either by analyzing individual prostate CTCs between patients with disease progression and enzalutamide naive group (P = 0.017, Fig. 3C), or by analysing AWE values for individual patients using all CTCs (P = 0.020, Fig. 3D) [87]. Thus, the observed AWE in CTCs could be clinically important as the quantitation of these values is correlated with the therapeutic outcomes at least for melanoma and prostate cancer. A comprehensive analysis including more single-cell RNA-seq datasets of CTCs generated from multiple cancer types is required to confirm the general clinical significance of AWE.

**Figure 4.**
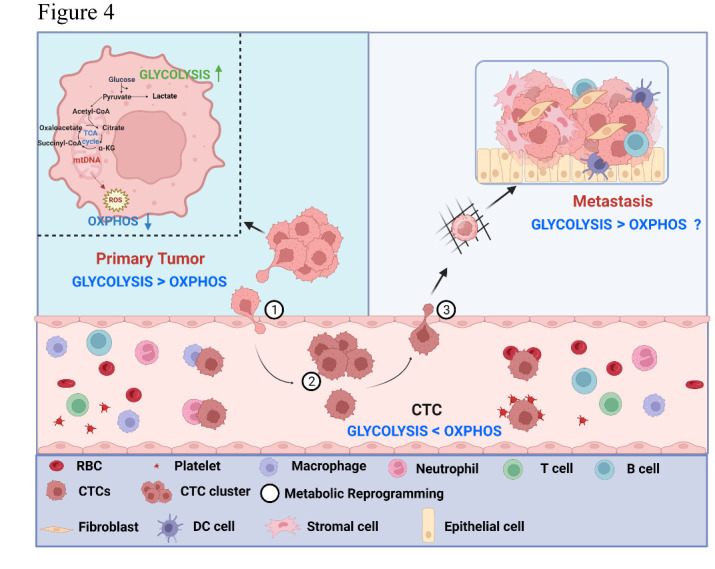
**The hypothetical model of CTC metabolic reprogramming during cancer metastasis**. In the primary tumor (Stage ①), cancer cells may employ the “Warburg Effect” to propel cell proliferation, where glycolytic flux overtakes OXPHOS. Upon transition to CTCs (Stage ②), a metabolic shift occurs, steering towards the “Anti-Warburg effect,” characterized by an intensified mitochondrial OXPHOS activity in relation to glycolysis. This shift may potentially support CTC survival within circulation and sustaining an optimal level of ROS may be critical for their optimal viability. Upon extravasation and arrival at distant metastatic sites (Stage ③), further metabolic reprogramming might take place, likely influenced by the intricacies of the new tumor microenvironment. This metabolic adaptation and flexibility may potentially contribute to successful colonigenic outgrowth.

Taken together, we hypothesized the existence of an intriguing phenomenon termed the "Anti-Warburg Effect" within CTC populations, a metabolic attribute seemingly at odds with conventional views, yet potentially harboring profound implications. Marked by the heightened OXPHOS relative to glycolysis expression signatures, the phenomenon of AWE suggests a potentially important metabolic switch in CTCs, which may contribute to their viability in the bloodstream and facilitate the enigmatic yet essential process during hematogenous dissemination of cancer cells (Fig. 4).

## DISCUSSION

Data from the publically available RNA-seq of single CTCs or cultured CTC cell lines in four cancer types suggests these tumor cells may exhibit remarkable metabolic flexibilities during distinct steps of metastasis. The transcriptomic analysis showed that when cancer cells detached from primary or metastatic deposits and enter the bloodstream to become CTCs, they unexpectedly elevated OXPHOS over glycolysis signatures, suggesting a metabolic reprogramming step that is potentially opposite to the common “Warburg Effect” seen in other metastatic cancer cells (Fig. 4). While the observed AWE may be a surprising phenomenon, we consider the following reasons that might account for this observation.

First, detachment of cancer cells may result in slow down of cell growth or cell cycle arrest, as evidenced by the fact that most CTCs do not proliferate or appear to be apoptotic [89, 90]. This might be caused by the shutdown or inactivation of oncogenic signaling pathways, such as EGFR, Ras, YAP1/TAZ and others, which could lead to reduced glycolysis and subsequently shift the metabolism towards mitochondrial oxidative metabolism [91, 92]. Hence, CTCs may intrinsically lack the ability to sustain elevated glycolytic flux and become heavily dependent on mitochondrial-mediated metabolic flux to maintain their viability in circulation. Although most cancer cells exhibit a restrained TCA cycle and oxidative phosphorylation, mitochondria remain a vital cellular resource for energy production and biosynthesis. Depending on the different cancer types, it was reported that up to 40-80% of total cellular ATP is generated by mitochondrial respiration [93]. The mitochondrial electron transportation chain has been shown to be essential for cancer cell proliferation [94-96]. Consistently, some cancer cells are critically dependent on mitochondrial activity upon shutdown of oncogenic signaling and eventually lead to tumor recurrence [97]. Therefore, elevated mitochondrial activity may be sufficient to support CTC survival in circulation, despite the inactivation of oncogenic signaling due to the loss in cell-cell and/or cell-matrix interactions.

Second, CTCs may require adequate levels of cellular ROS level to maintain their viability and metastatic potential in circulation. Indeed, it has been suggested that ROS may serve as a double-edged sword in cancer progression [98, 99]. ROS production at a certain threshold may serve as a potent second messenger to activate various oncogenic signaling pathways and support metastasis [100-102]. One prominent example is the identification of mtDNA mutations in the gene encoding NADH that confer metastasis in human tumors. These mutations lead to defective ETC complex I activity and increased ROS production. Pretreatment of ROS scavengers in these cancer cells damped their metastatic potential, suggesting a causal relationship between ROS and metastasis [103]. However, several other studies demonstrated an inhibitory function of heightened ROS levels in blood-borne metastasis in mouse cancer models [104-106]. Indeed, excess ROS production may lead to the activation of DNA damage checkpoint pathway and the eventual execution of programmed cell death programs in various cancer types [107, 108]. In agreement with this, it was found that the patient-derived breast CTCs have developed unconventional mechanisms to protect them from ROS damage [109].

Hence, it could be hypothesized that mitochondrial oxidative metabolism not only may sustain the survival of CTCs by providing vital energy and building blocks for biomass synthesis but also intricately shapes the metastatic diversity of CTCs via ROS-induced cellular fate choices. This process may exert an evolutionary selection pressure favoring the most well-suited surviving CTCs, effectively priming them for progression into the subsequent stages of the metastatic cascade.

Our analysis and ensuing hypothesis bear certain limitations that warrant rigorous validation. Given the pronounced heterogeneity in the growth and proliferation traits of metastatic tumor cells, it remains plausible that while some CTCs may lean towards oxidative phosphorylation, others might exhibit a preference for aerobic glycolysis. Indeed, investigations have unveiled glycolysis-related CTC signatures that exhibit correlations with clinical outcomes, as exemplified by Yang et al in their lung cancer study [110]. Moreover, it is worth mentioning that CTCs stemming from diverse cancer types could harbor distinct metabolic profiles. The systematic examination of larger cohorts of patient-derived CTCs across various cancer types remains to be carried out, a crucial endeavor to affirm their genetic and metabolic attributes using various single-cell omic technologies.

The present analysis draws exclusively from CTC transcriptomic datasets, leaving a direct link to metabolism yet to be established. The emergence of other omic technologies, like mass spectrometry-based single-cell metabolomic profiling methods, may hold great promise in uncovering the metabolic characteristics of individual CTCs [111, 112]. Indeed, a comprehensive exploration into the metabolic reprogramming of CTCs across different cancer types may offer insights into the common metabolic regulatory mechanisms within distinct metastatic cascades. The revelation of patient-specific metabolic traits in CTCs could bring fresh perspectives concerning the evolutionary dynamics of cancer progression, paving the way for personalized therapeutic approaches targeting metastasis.

### Datasets selection criteria and method details for bioinformatic analysis

We conducted a search with following key words in GEO database (https://www.ncbi.nlm.nih.gov/gds/?term=): CTC[All Fields] OR circulating tumor cell[All Fields] AND "Expression profiling by high throughput sequencing"[Filter]. A total of 106 datasets were accessed in which 6 datasets were eligible for this study after filtering with following criteria: 1. Samples were collected from patients rather than animal models. 2. Containing RNA seq data of match CTCs and tumor tissues. 4. Data are of high quality with balanced feature counts between CTC and tumor tissues (Median feature counts > 1000). The gene reads count matrixes of CTCs and tumor tissues were downloaded from the GEO database (GSE157740, GSE113890, GSE198291, GSE67980, GSE111842), GNSA database (CNP0000095) and normalized as log-rank FPKM (Fragments Per Kilobase Million). The H (hallmark gene sets) library was accessed through Molecular Signatures Database (MSigDB) (www.gsea-msigdb.org/gsea/msigdb/human/genesets.jsp?collection=H). Enrichment scores of HALLMARK_ OXIDATIVE_ PHOSPHORYLATION and HALLMARK_ GLYCOLYSIS were calculated using R package GSVA with default options (method = ‘gsva’). The “Anti-Warburg Effect” (AWE) value was defined by the enhanced oxidative phosphorylation over glycolysis which quantified using their enrichment scores (enrichment score of HALLMARK_OXIDATIVE_ PHOSPHORYLATION minus enrichment score of HALLMARK _GLYCOLYSIS). For comparing AWE values among CTCs, primary tumors and/or metastases, the function stat_compare_means (method = ‘wilcox.test’) in R package ggplot2 was employed. Single-cell RNA sequencing data were processed with R package Seurat. Malignant cells were annotated with inferCNV and CopyKAT. R packages Seurat, ggplot2, RColorBrewer, ggpubr were used for visualization.
